# CLOE: Identification of putative functional relationships among genes by comparison of expression profiles between two species

**DOI:** 10.1186/1471-2105-5-179

**Published:** 2004-11-19

**Authors:** Maurizio Pellegrino, Paolo Provero, Lorenzo Silengo, Ferdinando Di Cunto

**Affiliations:** 1 Department of Genetics, Biology and Biochemistry, Via Santena 5 bis, 10126, Torino, Italy; 2 Fondazione per le Biotecnologie, Viale Settimio Severo, Torino, Italy

## Abstract

**Background:**

Public repositories of microarray data contain an incredible amount of information that is potentially relevant to explore functional relationships among genes by meta-analysis of expression profiles. However, the widespread use of this resource by the scientific community is at the moment limited by the limited availability of effective tools of analysis. We here describe CLOE, a simple cDNA microarray data mining strategy based on meta-analysis of datasets from pairs of species. The method consists in ranking EST probes in the datasets of the two species according to the similarity of their expression profiles with that of two EST probes from orthologous genes, and extracting orthologous EST pairs from a given top interval of the ranked lists. The Gene Ontology annotation of the obtained candidate partners is then analyzed for keywords overrepresentation.

**Results:**

We demonstrate the capabilities of the approach by testing its predictive power on three proteomically-defined mammalian protein complexes, in comparison with single and multiple species meta-analysis approaches. Our results show that CLOE can find candidate partners for a greater number of genes, if compared to multiple species co-expression analysis, but retains a comparable specificity even when applied to species as close as mouse and human. On the other hand, it is much more specific than single organisms co-expression analysis, strongly reducing the number of potential candidate partners for a given gene of interest.

**Conclusions:**

CLOE represents a simple and effective data mining approach that can be easily used for meta-analysis of cDNA microarray experiments characterized by very heterogeneous coverage. Importantly, it produces for genes of interest an average number of high confidence putative partners that is in the range of standard experimental validation techniques.

## Background

The availability of genome sequences from several model organisms, including humans, and of high-throughput technologies to study gene function is dramatically changing the approach to biological problems. In particular, the consolidated reductionist gene-by-gene strategy is being replaced by a 'modular approach', in which several genes are studied simultaneously to gather a more comprehensive picture of the many different cellular processes [1]: in living organisms, the majority of gene products are part of intricate molecular circuits, composed of physical, functional and regulatory interactions. In higher eukaryotes, the study of gene function is further complicated by the alternative use of transcriptional units, frequently resulting in the production of proteins with different or even antagonistic activities from the same genes [2,3].

It is well recognized that one of the most important and widespread mechanisms used by cells to regulate functional modules is the coordinate transcriptional and/or post-transcriptional modulation of mRNA levels of the interacting genes. Therefore, DNA microarrays represent a fundamental tool to unravel biological complexity on a genome-wide scale. Information concerning the expression of thousands of genes, and also of different transcripts from the same gene, can be obtained in a single experiment, and the relationships among gene expression patterns can be studied systematically [4]. The extensive use of this technology by hundreds laboratories has resulted in the production of an enormous amount of data, many of which have been deposited in public databases [5,6].

Besides being useful to other researchers to confirm the published results, the deposited datasets can be used as a substrate for new analysis, aimed at discovering functional modules by searching for related expression profiles. Recent studies have shown that, if the expression of two or more genes is consistently related throughout many independent microarray datasets, the genes display a significant degree of functional similarity [7,8]. However, if this approach were applied to predict physical and functional relationships, a very high number of false positives would still be expected. A first method that can be used to reduce the number of false positives is to consider only co-expression links that are consistent among many different experimental datasets [7]. Nevertheless, even when the co-expression of two genes is reproducibly observed under a certain number of experimental conditions, this does not imply necessarily that they are functionally related. For instance, extensive meta analysis of microarray data across different species has revealed that neighboring genes are likely to be co-expressed, even though they are not functionally related in any obvious manner [9,10].

Phylogenetic conservation has been recently proposed as a very strong criterion to identify functionally relevant co-expression links among genes [11]. Significant co-expression of two or more orthologous genes across many species is very likely due to selective advantages, strongly suggesting a functional relation. In fact, the comparison of data across species as distant as *Homo sapiens*, *Saccharomyces cerevisiae*, *Drosophila melanogaster *and *Caenorhabditis elegans *was very effective in identifying new genes involved in core biological functions [11]. Although extremely specific, this multi-species approach would be unable to identify the relationships among genes involved in more specialized biological processes.

Since regulatory regions diverge much more rapidly than coding sequences [12,13], a similar approach would be predicted to succeed even when comparing expression patterns in more closely related species, such as mice and humans. In this case, the possible loss of specificity would be strongly compensated by the increased sensitivity in the identification of functional links related to mammalian-specific gene modules. This possibility has not been so far explored.

Additionally, when using microarray data to establish significant correlations among gene expression profiles, almost invariably the information obtained with probes covering different gene portions is averaged [14]. Though useful in many cases to reduce the experimental noise, this procedure could result in a significant loss of information in the case of genes expressing different isoforms with distinct expression patterns [15]: on one hand the isoform-specific expression profiles would not be detected; on the other hand, the average expression profile would be artificial and non-informative.

In this study we describe CLOE (Coexpression-based Linking of Orthologous ESTs) a new data mining method for the identification of transcripts showing evolutionary conserved co-expression in cDNA microarray datasets. This approach is based on the pairwise comparison of data from two species. The predictive capability of the method was proved by comparing human with mouse data. Our results show that CLOE is a valuable tool for biologists that can be used to identify putative partners for genes of interest and/or to predict some of their functional properties.

## Results and discussion

### The top percentiles of expression similarity ranked lists obtained with human-mouse orthologous ESTs pairs are strongly enriched of orthologous ESTs

The aim of our method is to use the available microarray expression data to identify high- confidence putative partners for genes of interest.

The basic assumption is that, if two or more genes are part of a functional module, conserved between two species, they will be likely co-expressed in both species. In contrast, if the co-expression of two genes in one species has no functional meaning, it should not be conserved in the other.

**Figure 1:**
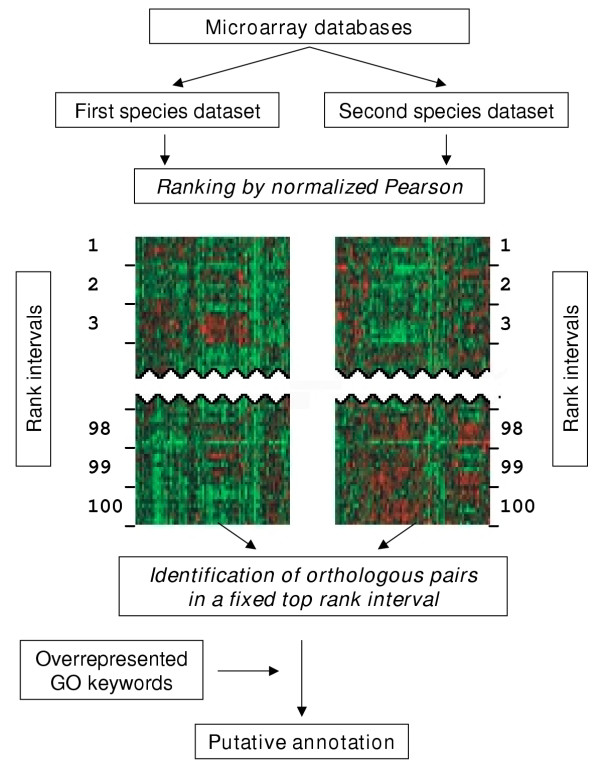
Schematic representation of the CLOE approach

A flow chart of the method is given in Figure 1. In summary, after finding representative EST clones for the gene of interest in cDNA microarray datasets of both species, we order all the ESTs in each dataset according to similarity of their expression pattern with that of the chosen ESTs. We then extract the orthologous pairs found in a given top percentage of the ranked lists. Moreover, to obtain a functional characterization of the identified putative partners, we analyze the co-expressed orthologous pairs for overrepresentation of Gene Ontology (GO) keywords [16].

Although in principle our method could be applied to every pair of organisms for which cDNA microarray data are available, we decided to compare the human and mouse datasets contained in the Stanford Microarray Database (SMD) [6] (2803 experiments for 74588 EST probes and 145 experiments for 37521 ESTs, respectively, data downloaded in Jan. 2004). The first reason for doing so is that this comparison is particularly relevant in the perspective of identifying mammalian-specific gene modules. The second is that, considering the widely different number of experiments and the relatively short phylogenetic distance between the two species, this represents a particularly severe test.

As a first proof of the method's effectiveness, and in order to empirically determine a reasonable default cutoff for obtaining the final list of candidates, we analyzed whether genes with the highest ranks in the single organism lists are actually enriched of orthologous sequences. To this aim, we randomly chose 100 orthologous gene pairs represented in both the human and mouse datasets and selected, for each one, the most representative EST (i.e. the probes with the highest number of experiments in each dataset). We then generated the respective ranked lists, subdivided them in 1% rank intervals and analyzed the number of orthologous pairs in corresponding rank intervals. As a control, we performed the same analysis on an equal number of randomly chosen (and hence non-orthologous) human-mouse EST pairs. The analysis was repeated three times with essentially identical results.

**Figure 2:**
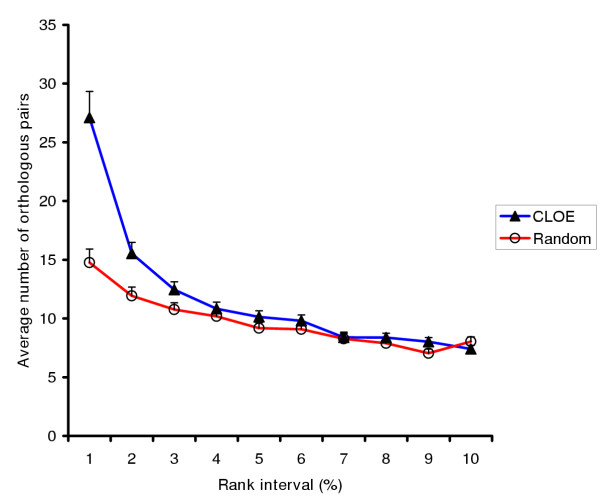
**The top percentiles of single organisms ranked lists obtained with orthologous probes are strongly enriched of orthologous sequences. **100 orthologous (CLOE) and 100 randomly chosen (Random) EST pairs were used to rank the ESTs in the human and mouse datasets on the basis of expression similarity. The ranked lists were divided in 1% rank intervals, and the average number of human ESTs in a given rank interval with at least one orthologous EST in the corresponding mouse rank interval was determined. The average number of these ESTs in the first top 10 rank intervals was plotted. Error bars = standard error.

As shown in Figure 2, compared to the control, a strong average enrichment of orthologous pairs was observed in the top 1% rank interval (p = 1.6·10^-94^, chi square test). The difference was still very significant in the 2% rank interval, even though with a much higher *p *value (p = 1.3·10^-10^), but was not detectable below that threshold. Interestingly, a slight enrichment was also observed in the last rank interval (average number of orthologous pairs equal to 7.8 for CLOE and 5.7 for random lists, p = 2.2·10^-7^). The latter observation is consistent with the previously noted fact that negative correlations tend to be less common and significant than positive correlations [7]. Based on these results, we chose a top 1% cutoff for all the following analysis.

### Predictive value of CLOE compared to single organisms and multiple species co-expression analysis

We next investigated the effectiveness of our approach, by comparing it to single and multiple organisms co-expression analysis. To address this point, we analyzed the ability of the three methods to predict known physical and functional interactions among mammalian proteins. Protein-protein complexes have begun to be determined on a genome-wide scale only for *Saccharomyces cerevisiae *[17], *Drosophila melanogaster *[18] and *Caenorhabditis elegans *[19], but no comparable datasets have been so far published for mammalians, making it impossible to perform a systematic comparison. Therefore, we focused on three supramolecular structures, which have been analyzed by different proteomic strategies at a high level of detail: the centrosome [20] (110 proteins), the post-synaptic density [21] (105 proteins) and the TNF-alpha/NFkB signalosome [22] (128 proteins). For the single organism and CLOE approaches, the analysis was restricted to proteins represented in the SMD by at least one human and one murine EST probe. These corresponded to 62, 67 and 97 ESTs pairs, respectively, covering on average 66% of the proteins found in these complexes. In contrast, only 37% of these genes were represented in the multiple species network, thus confirming that the previous two methods can be applied to a number of genes much higher than the latter.

The average number of candidates produced by CLOE for each analyzed protein was approximately 17, which represents a strong reduction if compared to the single organism approach (746 and 375 for the human and mouse datasets, respectively). On the other hand, the average percentage of CLOE links that correspond to a documented protein-protein interaction was 6.6 %, i.e. approximately 5 times higher than that obtained with the single organism method (Table 1). Significantly, the predictive value of human-mouse CLOE was very similar to that obtained by the multiple species co-expression network (Table 1).

Since considering only the proteomically-identified interactions could lead to a strong underestimation of the positive results, as low affinity and purely functional interactions would be completely excluded, we decided to evaluate the predictive power of the three different methods respect to a less stringent functional index. To this aim, we first determined which GO keywords represent the best annotation of the three complexes, by identifying the ones that are significantly overrepresented in the annotation of the respective proteins. Then, every predicted candidate partner obtained with the three methods for all analyzed proteins was considered as a true positive if it is annotated to at least one of the overrepresented keywords of the corresponding complex. The results of this analysis are summarized in Table 2.

Interestingly, even though also in this case our approach and the multiple species comparison gave, on average, a higher percentage of compatible predictions, this was not dramatically different from the single-organism method. These results strongly suggest that, compared to the single organism approach, the highly reduced number of candidate partners produced by multiple organism co-expression analysis and CLOE is strongly enriched of genes characterized by more stringent functional relationships.

## Conclusions

We have shown that CLOE represents a very flexible and effective data mining approach to infer a list of putative partners and the potential functions for genes of interest. It can be easily used for meta-analysis of cDNA microarray experiments characterized by very heterogeneous coverage, producing significant results even when data from two species as close as mouse and human are analyzed. Compared to single organisms co-expression analysis, it strongly reduces the number of potential partners for genes of interest, producing a list of targets that is highly enriched in physically interacting proteins. On the other hand, compared to multiple species co-expression analysis, it retains a comparable specificity, but can find candidate partners for a greater number of genes. Since the number of candidate partners obtained by this analysis is, on average, in the range of standard experimental validation techniques, we believe CLOE represents a useful tool for the exploration of gene function.

## Methods

### Definition of orthologous ESTs

The first step of our procedure is the identification of orthologous ESTs in the two datasets. Although many different methods could be used to this purpose, we relied on the InParanoid algorithm [23], which is ideally suited for the identification of orthologous sequences between two species. The results shown were obtained using the pre-computed release 2.6 of the InParanoid database [24]. ESTs were linked to InParanoid clusters through their UniProt codes [25,26], and their association to UniGene identifiers [27,28].

### Choice of representative probes for the gene of interest

The procedure has been implemented for the analysis of cDNA microarray datasets, such as those contained in the SMD [6,29]. However, it could be adapted, with minor modifications, to the analysis of Affymetrix datasets.

Ratiometric data for the different organisms are not subjected to any further normalization, and downloaded as log-transformed (base 2) ratios.

Within these datasets, the number of ESTs representing a given transcription unit, as well as the number of valid experiments for each EST, are highly variable. While this feature would pose serious problems, if one should attempt to average the data of all the probes belonging to the same gene, it may offer extremely valuable information when every EST is considered independently, since each clone explores the expression properties of a particular group of exons, in a particular combination of experimental situations.

Non-correlated or anti-correlated expression between two well-represented EST probes belonging to the same gene would strongly suggest that they correspond to alternatively expressed transcripts. For this reason, we decided not to merge the data of probes belonging to the same gene, but to treat separately every EST probe in the datasets.

The choice of the most representative EST for the genes of interest represents a particularly critical aspect of our procedure. If interested to a single gene, the more exhaustive solution to this problem would be to generate and evaluate a list of candidate partners for every possible pairwise combination of ESTs probes. However, this would greatly complicate the analysis should one be interested in analyzing the potential partners of many genes. An alternative possibility is to focus, for every Unigene cluster, on the most representative ESTs, i.e. the probes with the highest number of experiments. For these reasons, the decision about what ESTs to analyze is left to the end user.

Our implementation of the method can accept as input both a UniGene cluster ID or the results of a BLAST search performed with the sequence of interest against the EST database. In both cases, it retrieves a list of all probes found in the two datasets for the orthologous UniGene clusters.

To help the user decide which ESTs to analyze, the program provides basic information about all the EST probes representing the gene of interest in the two datasets. Moreover, to help the user identify the most representative EST probe in each dataset, i.e. the probe with the highest number of experiments, it also provides the number of valid data points for every probe. Finally, to assess the redundancy of the information provided by the different probes, i.e. whether they represent different experiments and display similar/different expression profiles, the program calculates, for every pair of probes belonging to the same UniGene cluster, the number of common experiments and the Pearson correlation coefficient between their expression profiles.

### Identification of orthologous sequences coexpressed in both species

After finding representative EST clones for the gene of interest in both species, we calculate, for each one, the Pearson correlation coefficient (*r*) with every other EST in the respective dataset. The raw *r *is the normalized for the number of common data points (*n*) obtained for the analyzed ESTs. This is done by multiplying *r *for 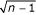, since the statistical significance of *r *is a function of the product 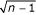
·*r*. Such normalization is particularly important when, as in our case, *n *has a very wide range of variation. The ESTs are then ranked by decreasing normalized score. Finally, a user-defined top percentage of the two ranked lists is compared to identify those ESTs that are associated to the same InParanoid cluster ID. A non-redundant list of the positive InParanoid clusters, sorted by the average highest rank obtained in the two organisms, represents the main output of the program (see Table 3 for an example). Clearly, the choice of the cutoff top rank percentage represents a critical parameter, which may strongly influence the number of identified candidates. The empirical determination of an average best cutoff is reported in the results.

### Functional characterization of the co-expressed orthologous clusters

After obtaining a list of putative partners for the genes under study, we analyze their functional characterization according to the GO vocabulary [16,30]. This is very useful to obtain new insight about the putative functional properties of the gene of interest. GO terms are associated to ESTs through the corresponding UniProt identifiers. For each list of candidates, we compute the prevalence of all GO terms among the annotated ESTs, and the probability that such prevalence would occur in a randomly chosen set of ESTs of the same size. We always consider a gene annotated to a GO term if it is directly annotated to it or to any of its descendants in the GO graph. For a given GO term t let K(t) be the total number of ESTs annotated to it in the first organism dataset that have an orthologous sequence in the second organism dataset, and k(m, t) the number of ESTs annotated to it in the final list S(m). If J and j(m) denote the number of orthologous ESTs in the dataset and in S(m) respectively, such probability is given by the right tail of the appropriate hypergeometric distribution:


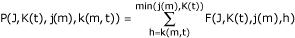


where


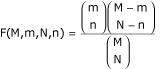


As an example, the results of the analysis performed on the output shown in Table 4. A similar strategy was used in all other cases where GO keywords overrepresentation test were performed.

### Availability

The programs used for this work are publicly available at the URL:

. This page contains the following files:

programs.zip (the program files and the corresponding General Public License);

readme.txt (detailed instructions for using our program).

## Authors' contributions

MP: development of most of the software, execution of the described analysis.

PP: development of the routines used to calculate the normalized Pearson, supervision of the statistical analysis.

LS: assessment of the biological significance of results.

FD: supervision of the project, manuscript writing.

## Figures and Tables

**Table 1:** Percentage of known protein-protein interactions in the lists of candidate partners generated by the different co-expression-based approaches. For every protein found in the three analyzed complexes, and represented by at least one EST probe in both datasets, we selected the most representative human and mouse probes. A CLOE analysis with a top 1% cutoff was performed on these sequences. In parallel, the human dataset was ranked for each human EST, and lists of candidates corresponding to the top 1% ranks where obtained (Single organism). The prevalence of ESTs corresponding to other proteins of the same complexes was then determined for both approaches. Finally, to determine the prevalence of correct predictions by the Multiple Species approach, we determined the ratio between the number of links with other proteins of the same complex and the total number of links for all the complex components (data from [11]).

	**Single organism**	**Multiple organisms**	**Human/mouse CLOE**
Centrosome	1.4	4.2	6.2
PSD	0.9	5.5	6.5
TNFα/NF-kB	1.6	6.1	6.8
Average	1.3	5.7	6.6

**Table 2:** Prevalence of functionally compatible predictions obtained with the three different methods. The percentage of compatible predictions was determined as in the previous table using the functional index described in the text.

	**Single organism**	**Multiple organisms**	**Human/Mouse CLOE**
Centrosome	19.5	36	26.3
PSD	33.8	47.8	41.3
TNFα/NF-kB	47.2	47.4	44.8
Average	33.5	43.7	37.4

**Table 3:** List of candidate partners generated by CLOE analysis on the most representative ESTs corresponding to the protein of unknown function FAD104.

**InParanoid**	**Human EST with highest rank**	**Human UniGene name**	**Rank**	**Mouse EST with highest rank**	**Mouse UniGene name**	**Rank**	**Average rank**
523	IMAGE:240295	FAD104	1	H3020H08	1600019O04Rik	1	1
1668	IMAGE:343072	ITGB1	2	IMAGE:1051975	Itgb1	45	23.5
9060	IMAGE:786680	ANXA5	21	H3016C05	Anxa5	103	62
8045	IMAGE:486787	CNN3	102	H3056D03	Cnn3	29	65.5
9769	IMAGE:488479	TPM1	107	3110002E24	Tpm1	57	82
11683	IMAGE:487437	PPIC	87	H3028H10	Ppic	93	90
6369	IMAGE:142788	SERPINH1	70	H3125A07	Serpinh1	129	99.5
899	IMAGE:469969	ITGAV	18	1110004F14	Itgav	182	100
9579	IMAGE:345538	CTSL	140	2600002C17	Ctsl	95	117.5
8192	IMAGE:613056	RCN1	52	H3027B09	Rcn	195	23.5
11615	IMAGE:230261	RALA	78	H3121E01	Rala	198	138
221	IMAGE:897760	LAMC1	43	H3113E11	Lamc1	239	141
1306	IMAGE:897164	CTNNA1	258	2210403L09	Catna1	48	153
4123	IMAGE:840697	FKBP9	83	H3147A05	Fkbp9	236	159.5
12331	IMAGE:841664	CAV1	24	H3089D06	Cav	301	162.5
5726	IMAGE:377384	NR2F2	308	H3124H07	Nr2f2	26	167
13914	IMAGE:810485	ID1	5	H3003F10	Idb1	365	185

**Table 4:** Gene Ontology keywords overrepresented in the list shown in the previous supplementary table. The results strongly suggest that this protein could be involved in some aspects of the functional interaction between the cytoskeleton and the extracellular matrix.

**Keyword**	**Organizing principle**	**p-value**
Endoplasmic reticulum	Cellular Component	9.3·10^-3^
Protein binding	Molecular Function	6.5·10^-3^
Peptidyl-prolyl cis-trans isomerase	Molecular Function	6.5·10^-3^
Structural constituent of muscle	Molecular Function	3.4·10^-3^
Collagen binding	Molecular Function	3.1·10^-3^
Structural molecule	Molecular Function	1.7·10^-3^
Tropomyosin binding	Molecular Function	8.9·10^-4^
Basement membrane	Cellular Component	5.7·10^-4^
Cytoskeleton	Cellular Component	5.6·10^-4^
Cell adhesion	Biological Process	6.4·10^-5^
Actin binding	Molecular Function	4.6·10^-8^
